# Influence of Surface-Modification via PEGylation or Chitosanization of Lipidic Nanocarriers on In Vivo Pharmacokinetic/Pharmacodynamic Profiles of Apixaban

**DOI:** 10.3390/pharmaceutics15061668

**Published:** 2023-06-07

**Authors:** Mohamed F. Zaky, Taha M. Hammady, Shadeed Gad, Abdullah Alattar, Reem Alshaman, Ann Hegazy, Sawsan A. Zaitone, Mamdouh Mostafa Ghorab, Mohamed A. Megahed

**Affiliations:** 1 Department of Pharmaceutics and Pharmaceutical Technology, Faculty of Pharmacy, Egyptian Russian University, Cairo 11829, Egypt; mohammed-fawzy@eru.edu.eg (M.F.Z.);; 2 Department of Pharmaceutics and Industrial Pharmacy, Faculty of Pharmacy, Suez Canal University, Ismailia 41522, Egypt; taha_hamadi@pharm.suez.edu.eg (T.M.H.);; 3 Department of Pharmacology & Toxicology, Faculty of Pharmacy, University of Tabuk, Tabuk 71491, Saudi Arabia; aalattar@ut.edu.sa (A.A.); ralshaman@ut.edu.sa (R.A.); szaitone@ut.edu.sa (S.A.Z.); 4 Department of Clinical Pathology, Faculty of Medicine, Suez Canal University, Ismailia 41522, Egypt; ann_hegazy@med.suez.edu.eg; 5 Department of Pharmacology and Toxicology, Faculty of Pharmacy, Suez Canal University, Ismailia 41522, Egypt

**Keywords:** apixaban, nanostructured lipid carriers, chitosanization, PEGylation, pharmacokinetics/pharmacodynamics

## Abstract

Nanostructured lipid carriers (NLCs) have been proven to significantly improve the bioavailability and efficacy of many drugs; however, they still have many limitations. These limitations could hinder their potential for enhancing the bioavailability of poorly water-soluble drugs and, therefore, require further amendments. From this perspective, we have investigated how the chitosanization and PEGylation of NLCs affected their ability to function as a delivery system for apixaban (APX). These surface modifications could enhance the ability of NLCs to improve the bioavailability and pharmacodynamic activity of the loaded drug. In vitro and in vivo studies were carried out to examine APX-loaded NLCs, chitosan-modified NLCs, and PEGylated NLCs. The three nanoarchitectures displayed a Higuchi-diffusion release pattern in vitro, in addition to having their vesicular outline proven via electron microscopy. PEGylated and chitosanized NLCs retained good stability over 3 months, versus the nonPEGylated and nonchitosanized NLCs. Interestingly, APX-loaded chitosan-modified NLCs displayed better stability than the APX-loaded PEGylated NLCs, in terms of mean vesicle size after 90 days. On the other hand, the absorption profile of APX (AUC_0-inf_) in rats pretreated with APX-loaded PEGylated NLCs (108.59 µg·mL^−1^·h^−1^) was significantly higher than the AUC_0-inf_ of APX in rats pretreated with APX-loaded chitosan-modified NLCs (93.397 µg·mL^−1^·h^−1^), and both were also significantly higher than AUC_0-inf_ of APX-Loaded NLCs (55.435 µg·mL^−1^·h^−1^). Chitosan-coated NLCs enhanced APX anticoagulant activity with increased prothrombin time and activated partial thromboplastin time by 1.6- and 1.55-folds, respectively, compared to unmodified NLCs, and by 1.23- and 1.37-folds, respectively, compared to PEGylated NLCs. The PEGylation and chitosanization of NLCs enhanced the bioavailability and anticoagulant activity of APX over the nonmodified NLCs; this highlighted the importance of both approaches.

## 1. Introduction

Globally, venous thromboembolism (VTE) is considered one of the leading causes of morbidity and mortality. A notable portion of VTE burden in home-cured and hospitalized patients is caused by pulmonary embolism and deep vein thrombosis [1,2]. Until 10 years ago, medications such as warfarin, fondaparinux, low molecular weight heparin, and unfractionated heparin were the only alternatives for treating VTE. Due to the variety of medications that patients are exposed to, the limited therapeutic window, the need for frequent coagulation testing, and the numerous drug–drug and drug–food interactions, patients are more likely to be less adherent to their treatment regimens [3]. Most of the aforementioned limitations have been eliminated since the elaboration of direct-acting oral anticoagulants (DOACs) for the treatment and secondary prophylaxis of VTE [4]. 

Apixaban (APX) is an effective DOAC medication applied clinically to prevent VTEs following total knee or hip replacement surgeries [5]. By blocking the factor Xa in both its free and clot-bound forms, APX inhibits thrombin synthesis and thrombus development [6]. APX is administered in a 2.5 mg dose twice daily (which can be increased to 5 mg BID) in order to treat VTEs [7]. Unfortunately, it has poor aqueous solubility (0.028 mg/mL) and limited oral bioavailability, possibly due to first-pass metabolism in the gastrointestinal tract and the liver, as well as to noncomplete absorption through the gut [8,9]. In addition, APX suffers from a potential for bleeding, an obvious adverse effect following multiple drug administration [10,11]. Hence, there is a crucial need for developing new APX formulations that can boost its solubility and enhance its bioavailability.

The biocompatibility, biodegradability, and diversity of biomedical applications for lipid-based nanocarriers have increased our interest in researching these nanovesicles [12]. Such nanocarriers have an added benefit over other nanosystems used for drug delivery; the lipids utilized in their structure can potentially protect the encapsulated drug particles from enzymatic, oxidative, chemical, and electromagnetic decomposition [13]. Originally, lipidic nanoparticles were formulated as solid lipid nanoparticles (SLNs), but later, a second generation was developed, namely nanostructured lipid carriers (NLCs). The latter differs from SLNs in that a liquid lipid replaces part of the solid lipid, thus disrupting the solid/mass structure of SLNs and providing extra spaces for greater incorporation of the active pharmaceutical ingredient (API) [14]. The structure and composition of NLCs increased drug loading and improved the formulation’s stability during storage [15]. Additionally, several drugs with poor bioavailability were fabricated in the form of NLCs, and subsequently found to have a significant enhancement of their oral bioavailability. This was evidenced by an increased area under the curve of their NLC formulations compared to drugs’ suspensions; as the increase was by 7.2-, 7.5-, 8.3-, 16.5-, and 26.3-folds for Tacrolimus [16], docetaxel [17], iloperidone [18], lopinavir [19], and nintedanib esylate [20], respectively.

Notwithstanding the benefits, NLCs’ applicability and marketability have been constrained by various issues, such as lipid modification, separation of the lipid phase, and high coagulation rate [21]. Although some reports stated that few NLC formulations could escape the uptake by the reticuloendothelial system (RES), it was also observed that other NLCs might be quickly absorbed by the RES and transported out of the vascular system to the liver, spleen, or bone marrow. Additionally, this RES accumulation frequently results in NLCs’ toxicity [22]. These flaws are the major obstacles for our research into designing and utilizing NLCs to deliver medications.

Interestingly, using various polymeric coatings, the performance of the conventional NLCs can be more efficiently enhanced. In addition, these coatings can improve their mucoadhesive property, which increases their absorption rate and delivery effectiveness. They may also increase the API chemical and enzymatic stability, decrease drug loss over storage time, enhance colloidal stability, and increase drug solubility [23]. Furthermore, a well-known method for increasing the oral bioavailability of poorly soluble medications is to develop a modified NLC formulation coated with certain polymers, such as polyethylene glycol (PEG) and/or chitosan [24,25].

Owing to its safety, biocompatibility, and biodegradability, in addition to its potency as a thickening, gelling, and antibacterial agent, chitosan has been employed extensively in many pharmaceutical and food industries [26]. Chitosan has mucoadhesive properties, greatly improving drug absorption by coating the nanocarrier with this polymer. For hydrophobic medications, chitosan can increase their solubility/dissolution rate, their bioavailability, and their penetration into the gastrointestinal wall [27]. This polymer is widely employed in studies on new drug delivery systems. It has excellent benefits and comprehensive applications for various medication delivery methods, including oral, parenteral, topical, transdermal, vaginal, and rectal drug delivery [28,29].

Polyethylene glycol (PEG) is a nontoxic, nonirritating, inert hydrophilic polymer conjugated on the nanocarriers’ surfaces. The steric barriers that the PEG chains provide against plasma protein binding increase the stability of nanodrug delivery systems. This protection improves biodistribution and residence time at the site of action, which eventually enhances the pharmacokinetics and pharmacodynamics of drugs loaded in nanocarriers [22,30,31].

To sum up, the drawbacks of NLCs can be significantly reduced or even abolished via the utilization of a PEGylation or chitosanization approach, which can in turn further augment their pharmacokinetic/pharmacodynamic profiles. Therefore, this study aimed to examine how NLCs’ ability to function as an oral delivery system for APX was affected by the chitosan and PEG coating, so as to enhance its bioavailability and anticoagulant activity. The formulations under investigation were elaborated, fully characterized, and investigated through in vitro and in vivo studies, which have been described in detail in the following sections.

## 2. Materials and Methods

### 2.1. Materials

Chitosan (high purity, low molecular weight) and polyethylene glycol 4000 (PEG-4000) (for synthesis) were purchased from Sigma-Aldrich Co. (St. Louis, MO, USA). Apixaban (100 ± 0.5%) was gifted from Apex Pharma (Badr City, Egypt). Oleic acid (extrapure) was kindly gifted from Egyptian International Pharmaceuticals Industries Company (EIPICO) (10th of Ramadan City, Egypt). Stearic acid (98%), Tween 80 (99.8%), and potassium phosphate dibasic anhydrous (K_2_HPO_4_) (98%) were bought from Loba Chemie (Mumbai, India). Sodium lauryl sulphate (SLS) (95%) was purchased from Research Lab Fine Chemicals (Mumbai, India). Orthophosphoric acid (98%) was bought from Biochem (Cairo, Egypt). Acetonitrile HPLC grade was obtained from Merck (Darmstadt, Germany). Chloroform was obtained from Panreac Quimica SA (Barcelona, Spain). Methanol absolute, ChromAR^®^ HPLC was purchased from Macron fine chemicals (Center Valley, PA, USA). Lecithin (90%) was bought from Alfa Aesar (Haverhill, MA, USA).

### 2.2. Methods

#### 2.2.1. Development of APX-Loaded NLCs (APX-NLC) and APX-Loaded PEGylated NLCs (APX-PEG-NLC)

With added adjustments, thin film hydration (followed by ultrasonication) method was used to elaborate both drug-free NLCs and APX-NLC [32,33]. The specified amount of each ingredient was obtained following a previous study from our laboratory [34]. In short, stearic acid, oleic acid, and lecithin were weighed (100 mg, 100 mg, and 75 mg, respectively). Regarding the development of APX-PEG-NLC and drug-free PEGylated NLCs, PEG-4000 (20% *w*/*w* of the lipidic matrix, and 0.275% *w*/*v* of the total NLC dispersion) was added to the lipid mix [35,36]. Precisely 10 mg of APX was then added to the formed mix, which was subsequently added to a bear-shaped flask of a Büchi-M/HB-140 rotary evaporator (Gallen, Switzerland). With the aid of sonication, the obtained mixture was completely solubilized in a 2:1 chloroform–methanol mixture. The organic solvents were allowed to evaporate slowly and completely in a water bath (60 °C, 30 min) with the application of a vacuum till the production of a thin, dry film on the walls of the flask.

Next, 300 mg of Tween 80 was dissolved in deionized water (20 mL), to serve as the aqueous phase for the hydration of the precipitated film in a water bath (60 °C) for 60 min. About 10 glass beads, 4 mm in diameter, were added to produce complete hydration. The resultant dispersion was sonicated for 4 min utilizing a Vibra cell VCX 750 probe sonicator from Sonic and Materials Inc. (Newtown, CT, USA) at an amplitude of 40%, then stored at −20 °C till subsequent evaluations [37]. To develop blank nanovesicles, the same methodology was applied without incorporating APX in the formulations.

#### 2.2.2. Elaboration of APX-Loaded Chitosan-Modified NLCs (APX-Ch-NLC)

A transparent chitosan solution (with a 0.5% *w*/*v* concentration) was obtained by dissolving 1 g of chitosan in 200 mL of aqueous 3% acetic acid solution. The resultant solution was then agitated using a magnetic stirrer for 24 h at 25 °C before being meticulously filtered. In order to develop the APX-Ch-NLC, 10 mL of the prepared chitosan solution was added to the previously constructed APX-NLC, in addition to enough acetic acid to achieve a final chitosan concentration of 0.25% *w*/*v* of the total NLC dispersion and a pH value between 3.5 and 4.5 [38]. The same technique was used to develop blank nanovesicles, merely without including APX in the formulations. The generated nanovesicles were stored at −20 °C until further evaluation.

**Figure 1:**
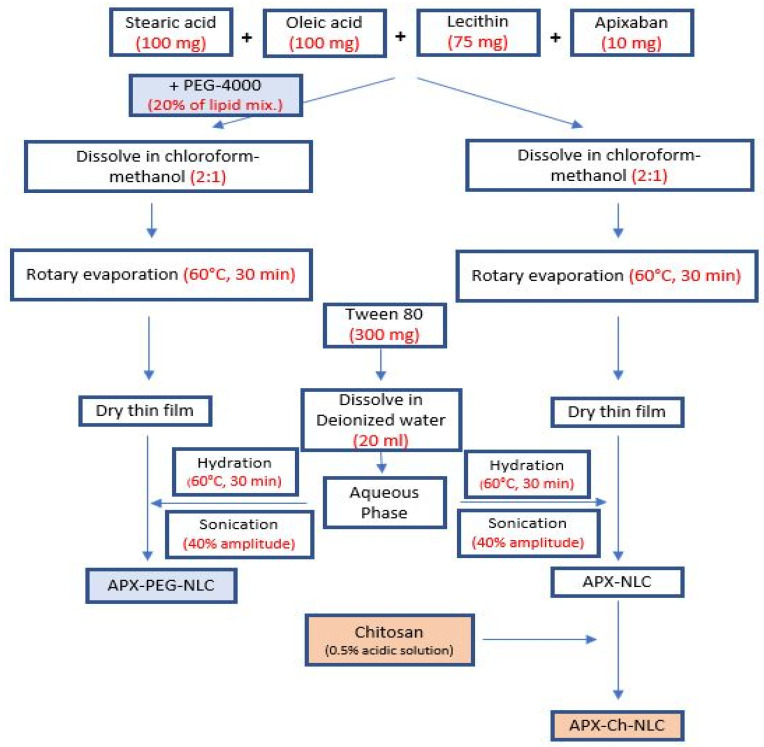
Flow chart for the preparation of unmodified and/or surface modified APX-loaded nanovesicles.

A flow chart summarizing the preparation techniques of APX-NLC, APX-PEG-NLC, and APX-Ch-NLC is presented in Figure 1.

#### 2.2.3. Separation and Washing of APX-Loaded Nanovesicles

At 65 °C, the frozen NLC formulations were thawed, utilizing a High-Speed Refrigerated Sigma Centrifuge (3–30 KS) from SIGMA Laborzentrifugen (Osterode, Germany) at −4 °C and a speed of 20,000 rpm for 2 h, separating both the free drug as well as unbound polymers from the NLC dispersions. Afterward, with the aid of a vortex mixer, NLC formulations were washed through redispersion in deionized water, before being centrifuged for a second cycle. This procedure was repeated to ensure the complete absence of free drug and unbound polymers from the voids between NLC vesicles [39,40,41].

#### 2.2.4. Characterization of APX-Loaded Nanovesicles

##### Particle Size and Zeta Potential Evaluation 

Before assays, all the manipulated nanoformulations were sonicated for 5 min in order to break up any clumps and remove air after (1/10) dilution with deionized water [42]. Mean particle size (intensity averaged, and measured in nm), polydispersity index (PDI), and zeta potential (ZP, measured in mV) for all developed nanovesicles were evaluated at 25 °C with dynamic light scattering (DLS) with laser diffraction using Nicomp^TM^ 380/ZLS Particle sizing/ZP system (Santa Barbara, CA, USA). The assessment was carried out three times, then the average reading served as the mean vesicle size and ZP.

##### Entrapment Efficiency (EE%) Determination 

Entrapment efficiency was assessed by draining precisely 1 mL of the washed NLC dispersions using a 100–1000 μL micropipette. Then, methanol was added to burst the particles and release the entrapped drug, and the final volume was subsequently adjusted to 10 mL. Afterward, the solution was sonicated until it became clear, before being spectrophotometrically analyzed at 279 nm wavelength utilizing an UV spectrophotometer, namely, the Jasco V-630 (Tokyo, Japan) [43], with drug-free NLCs serving as a control. The measurements were carried out three times, after which Equation (1) was applied to calculate the EE% [44]:

(1) $$\mathrm{EE}\%=\frac{\mathrm{Amount}\;\mathrm{of}\;\mathrm{entrapped}\;\mathrm{Apx}}{\mathrm{Total}\;\mathrm{amount}\;\mathrm{of}\;\mathrm{Apx}\;\mathrm{added}\;\mathrm{in}\;\mathrm{the}\;\mathrm{formulation}}\times 100$$

##### In Vitro APX Release Study and Mathematical Modeling of the Elaborated Nanovesicles 

The in vitro release properties of APX from all drug-loaded nanovesicles and free drug solution were investigated using the dialysis bag diffusion method [45]. A fixed 3 mL volume was taken from each preparation and filled in a dialysis bag of VISKING^®^ Dialysis Tubing type (MWCO 12,000–14,000) with a length of 4 cm and a diameter of 2.1 cm. Phosphate buffer solution (PBS) was applied as the release medium, with the addition of 0.05% SLS to achieve sink conditions and enhance drug solubility [46]. The pH of the solution was adjusted to 6.8 with the help of dipotassium hydrogen phosphate and orthophosphoric acid. The study was conducted utilizing a Daihan ThermoStable^TM^ IS-20 benchtop shaking incubator (Wonju, Republic of Korea). The temperature was stabilized at 37 °C, and the shaker speed was maintained at 140 rpm. Samples (1 mL) were taken from the release medium at preset time points (0.5, 1, 2, 4, 6, 8, 12, and 24 h) and immediately replaced with fresh medium. Applying the same spectrophotometric analysis conditions described earlier, the amount of APX released was determined, with drug-free nanocarriers serving as the control. The studies were performed in triplicate, and the obtained results were used to construct the in vitro release graph for the cumulative % released over a 24 h period.

The release behaviors of APX from the developed nanovesicles were fitted to zero order (Equation (2)), first order (Equation (3)), second order (Equation (4)), Baker–Lonsdale model (Equation (5)), Hixon–Crowell release (Equation (6)), and Higuchi diffusion (Equation (7)) with the help of DDsolver, an excel based add-in program. They were then quantitatively matched to the release study data by comparing the Pearson’s correlation coefficients (r), selecting the model with the highest (r) value as the best fit. Moreover, the Korsmeyer–Peppas kinetic model (Equation (8)) was applied to confirm the release mechanisms [47,48].

m_t_ = m_b_ + k_0_t (2)

ln (m_0_ − m_t_) = ln (m_0_) − k_1_t (3)

(4) $$\frac{1}{\left(m_{0}-m_{t}\right)}=\frac{1}{m_{0}}-k_{2}t$$

3/2[1 − (1 − m_t_/m_0_)^2/3^]m_t_/m_0_ = k_3_t (5)

m_0_^1/3^ − m_left_^1/3^ = k_4_t (6)

m_t_ = k_H_t^0.5^ (7)

m_t_/m_∞_ = k_kp_·t^n^ (8)

where m_t_ is the amount of the drug released over time t; m_b_ is the amount of the drug in solution before release; m_0_ is the amount of the drug in the formulation at time 0; m_left_ is the amount of the drug left in the formulation after time t; $m_{\infty }$ is the amount of the drug released after an indefinite time (24 h in our research); k_1_, k_2_, k_3_, k_4_, k_H_, and k_kp_ are the release rate constants of zero order, first order, second order, Baker–Lonsdale model, Hixon–Crowell model, Higuchi diffusion, and Korsmeyer–Peppas model, respectively; and n is the diffusional exponent in the Korsmeyer–Peppas model.

##### Transmission Electron Microscopy (TEM) 

The generated nanoformulations were examined with a Jeol Jem-2100 TEM instrument (Tokyo, Japan) to confirm the obtained size and analyze the particle morphology. The formulations where then significantly diluted with deionized water to the proper intensity, enabling clear visualization of the created NLCs. One drop of the formed NLCs suspension was put into a carbon-coated grid, and the particles were allowed to stick to the grid by leaving them for one minute. A filter-paper sheet was applied to remove the extra suspension. After applying a drop of 1% phosphotungstic acid staining solution, the leftover stain was removed using the filter paper sheet again. Then, the samples were allowed to dry and subsequently inspected under an electron microscope [49].

##### Differential Scanning Calorimetry (DSC) 

DSC was applied to investigate the thermal behavior and interaction between APX and the lipidic-based nanovesicle compositions. A differential scanning calorimeter, DSC 60, Shimadzu (Kyoto, Japan), was utilized for the thermal analysis studies. A defined amount (about 3–5 mg) of the sample was put in an aluminum pan. The pan was placed into the device after being crimped. An empty pan was crimped similarly and served as a reference. The sample was heated at a constant rate of 10 °C/min for thermal analysis, which covered the temperature range of 25 to 300 °C. Nitrogen gas was continuously blown during the heating process. Thermal analysis software (TA-60WS) was used to track and analyze thermal behavior [50]. The DSC thermograms were recorded for APX, PEG-4000, Chitosan, Blank-NLC, APX-NLC, APX-PEG-NLC, and APX-Ch-NLC.

#### 2.2.5. Stability Study

The stability of the prepared nanovesicles was conducted to evaluate the effects of PEGylation and chitosan coating on the stability of APX-NLC. The APX-loaded nanoformulations were stored at 4 °C, and samples were withdrawn on Day 0, Day 30, Day 60, and Day 90 to determine mean vesicle diameter, ZP, and entrapment efficiency using the methods described above.

#### 2.2.6. In Vivo Evaluation of the Prepared Nanovesicles

##### Protocol and Animal Preparation

The protocol (#202104PHDA1), which had been authorized by the committee of research ethics, Faculty of Pharmacy, Suez Canal University, was followed for carrying out the animal studies. Forty-two male Wistar rats weighing 220 ± 20 g, with ages of 7–8 weeks, were used in the in vivo studies. During the investigation, the animals were kept in cages with a plastic mesh under normal humidity, temperature, and illumination (12 h light/dark cycles), in addition to being given free access to standard laboratory food and water. The day before conducting the experiment, all rats were deprived of food and water overnight. The rats were randomly assigned into one of the five experimental groups; Group A (6 rats) was administered normal saline and acted as a control group, and Groups B, C, D, and E (9 per group) were given a 60 mg/kg single oral dose of a free APX suspension, APX-NLC, APX-PEG-NLC, and APX-Ch-NLC, respectively [51]. Free drug suspension was prepared using the dispersion of APX in deionized water with the help of 0.2% gum tragacanth and glycerin [52]. The groups were subsequently split up again to conduct pharmacokinetic (PK) and pharmacodynamic experiments. Results were displayed as mean ± standard deviation (SD), and a *p*-value < 0.05 was deemed significant.

##### Pharmacokinetics Study

- Chromatographic conditions

Drug-plasma concentrations in the studied animals were analyzed utilizing a Waters Alliance e2695 reverse-phase high-performance liquid chromatography system (Milford, MA, USA), equipped with a PDA detector and a C18 column (250 × 4.6 mm, 5 µm) of Agilent ZORBAX Eclipse type (Agilent, CA, USA). The mobile phase employed consisted of a mixture of 50:50 (*v*/*v*) methanol:buffer solution (0.1% phosphoric acid in water). The flow rate was adjusted to be 1 mL/min, and the injection volume was 100 µL. The peaks were observed at 280 nm, and the temperature of the column and the total run time were 30 °C and 6 min, respectively [53,54].

- Pharmacokinetics (PK)

To conduct the pharmacokinetics study, 3 rats (weighing 220 ± 20 g, with ages of 7–8 weeks) from each study group (B, C, D, and E) were selected. At predefined time points (0.25, 0.5, 1, 2, 3, 4, 6, 8, 12, and 24 h), 0.5 mL of blood was withdrawn from the retro-orbital plexus into a sampling tube that contained 1/10 volume of 3.8% sodium citrate. To obtain plasma, a Microfuge E centrifuge from Beckman Instruments (Palo Alto, CA, USA) was utilized, and samples were centrifuged at 10,000 rpm for 5 min. The obtained plasma samples were frozen at −80 °C using an WUF-25 (Daihan Scientific Co., Ltd.) ultralow temperature freezer (Seoul, Republic of Korea). Then, at the time of analysis, 100 µL of the plasma samples were mixed with 50 µL of the internal standard (rivaroxaban 0.01 µg/mL) and 500 µL of acetonitrile. The obtained mixture was centrifuged at 5000 rpm for 20 min, then 100 µL of the supernatant was used for HPLC analysis [34]. Different PK parameters were measured using PK solver 2.0 software, and the data were presented as mean ± SD.

##### Pharmacodynamics (PD) Study

- Cuticle Bleeding Time (CBT)

CBT test was conducted using 15 rats (3 from each experimental group). The toenail of the animals’ hind paws was cut where the nail met the quick, using a single-edged razor blade, before being immediately immersed in Ringer’s solution at 37 °C. Maximum bleeding was defined as the time after the operator used external pressure to halt the bleeding [55]. While observing with a binocular microscope, with 3-times magnification, the time until the cessation of the bleeding (without rebleeding within 30 s) was calculated.

- Prothrombin Time (PT) and Activated Partial Thromboplastin Time (APTT)

Clotting time measures, such as PT and APTT, were evaluated to identify the effect of the drug-loaded nanoformulations on the coagulation cascade in contrast to the free APX suspension and control groups [56,57]. Blood samples from 3 rats (for each study group) were obtained 120 min after drug administration. The plasma was obtained as previously described in the PK section, before then being immediately utilized to assess PT and APTT. The Dade Thromboplastin-C reagent, obtained from the Baxter Healthcare Corporation (Deerfield, IL, USA) was utilized to assess PT using the technique of mechanical clot detection with an International Sensitivity Index (ISI) of 2.0. By contrast, APTT was assessed using both the mechanical clot detection technique and the Siemens Healthineers Dade^®^ Actin^®^ FSL reagent (Erlangen, Germany) [58].

#### 2.2.7. Statistical Analysis

GraphPad Prism 8 software from GraphPad Inc. (San Diego, CA, USA) was utilized to perform all statistical analyses. In vitro drug release, stability study, and in vivo plasma data were compared among different formulations using a two-way ANOVA with Tukey’s multiple comparisons test. Regarding the comparison of PK parameters, an unpaired student t-test was used. Finally, a one-way ANOVA/Tukey–Kramer post hoc test was applied to compare the pharmacodynamic parameters between all study groups. Results were deemed significant if the *p*-value was less than 0.05.

## 3. Results and Discussion

### 3.1. Characterization of the APX-Loaded Nanovesicles

#### 3.1.1. Particle Size and Zeta Potential

The stability and effectiveness of nanovesicles depended significantly on their size, PDI, and ZP [59]. As a result, these parameters were assessed and considered to be critical formulation parameters for the elaborated NLCs. Table 1 and Figures S1–S3 display the mean vesicle diameter and PDI values of all APX-loaded nanocarriers. Sizes of all studied formulations ranged from 267.4 ± 5.5 nm for APX-NLC to 403.4 ± 11.5 nm for APX-Ch-NLC. Additionally, PDI ranged from 0.21 for APX-PEG-NLC to 0.4 for APX-NLC, which proved the homogenous particle size distribution for all NLC formulations. The analysis of these results revealed that modifying NLCs with either PEG-4000 or chitosan led to increased particle size but reduced the PDI, reflecting the uniform size distribution of modified nanovesicles compared to conventional NLCs [60].

Regarding ZP, data presented in Table 1 showed the high stability of all NLC formulations (i.e., below −30 mV or above +30 mV) [61]. The data demonstrated that PEGylation of NLC decreased the ZP magnitude from −38.7 ± 2.2 mV to −34.2 ± 4.4 mV. This is in concordance with previously published reports, which revealed that the ethylene oxide chains of PEG reduced the surface charge of the particles by shifting the shear plane to the aqueous phase, which eventually results in lowering the value of ZP regardless of the charge of the nanocarriers [22,62]. Coating the nanovesicles with chitosan (a cationic polymer) shifted the ZP values from negative to positive, as displayed from the ZP of APX-Ch-NLC being 39.7 ± 1.5 mV [63].

#### 3.1.2. Entrapment Efficiency

As presented in Table 1, EE% was 85.1 ± 4.1% and 90.8 ± 6.2% for APX-NLC and APX-PEG-NLC, respectively, proving that the PEGylation increased the drug entrapment compared to the conventional NLCs. According to previous reports, the increased stability and tight walls created with the addition of PEG to the constructed nanovesicles may have caused the PEGylated NLCs’ higher drug encapsulation percentage [60]. Additionally, the physical exclusion of the encapsulated drug was prevented due to the presence of the PEG layer [21]. Table 1 shows that coating nanocarriers with chitosan enhances drug encapsulation. This enhancement could be attributed to larger particles allowing more API to be encapsulated in the core or nanovesicle matrix [64]. Moreover, electrostatic interaction between the positively charged chitosan coat and the negatively charged surface of NLCs might have led to more stabilized nanovesicles with minimal drug leakage [65].

#### 3.1.3. In Vitro Drug Release

**Figure 2:**
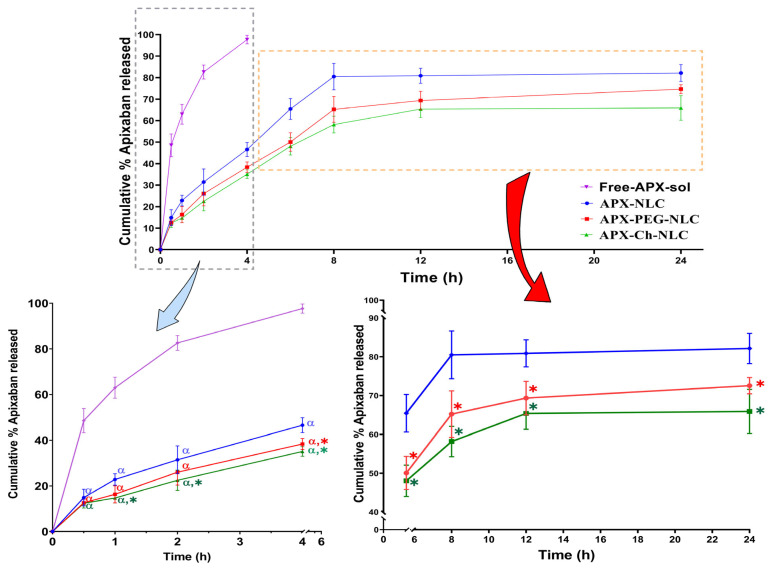
In vitro release profiles of Free APX drug solution, APX-loaded NLC, APX-PEG-NLC, and APX-Ch-NLC, plotted in terms of the cumulative percent released vs. time; data are displayed as mean ± SD, (n = 3). Note: * and α denote significant differences (at *p* < 0.05) from Free APX drug solution and APX-NLCs, respectively.

In general, in order to imitate a drug’s in vivo performance, it is crucial to understand the release profile of the drug from nanovesicles. As shown in Table 1 and Figure 2, the release study revealed that APX-NLC exhibited the highest release percent of APX after 24 h, with 82.1 ± 3.9%. At the same time, a lower % of the released drug was observed for APX-PEG-NLC and APX-Ch-NLC, being 72.6 ± 2.11% and 65.9 ± 5.7%, respectively. It was clear that the release profiles from all nanovesicles exhibited two phases: an initial rapid release phase due to the desorption of surface-attached drug particles, followed by a prolonged release phase owing to the diffusion of the drug from formulated nanocarriers [66]. Statistical analysis showed a significant difference (*p* < 0.05) between all APX-loaded nanovesicles and the free drug solution, which released almost 100% of the drug within 4 h. It also revealed a significant difference in drug release between unmodified and modified NLCs. This could be due to the inhibitory effects exerted by the PEG and chitosan coatings on the APX release from nanovesicles [60]. Interestingly, there was a nonsignificant difference (*p* > 0.05) between the cumulative release profiles of APX-PEG-NLC and APX-Ch-NLC. 

To forecast the release mechanisms associated with all NLC formulations, mathematical models were used. APX release from all nanovesicles was found to follow the Higuchi release kinetics, which exhibited the highest correlation coefficient (r) value, as presented in Table 2. The release rate constants for different models are displayed in Table S1. The diffusional exponent (n) in the Korsmeyer–Peppas equation was found to be <0.5 in the case of APX-NLC, indicating the Fickian diffusion release mechanism (Table S2). Contrastingly, it was >0.5 for both APX-PEG-NLC and APX-Ch-NLC with anomalous (non-Fickian) release mechanism, indicating that release from coated NLCs was governed by the combined diffusion from the matrix as well as its erosion. These results agree with the previously published data for the release mechanism in conventional and modified NLC formulations [67,68,69].

#### 3.1.4. Transmission Electron Microscope 

**Figure 3:**
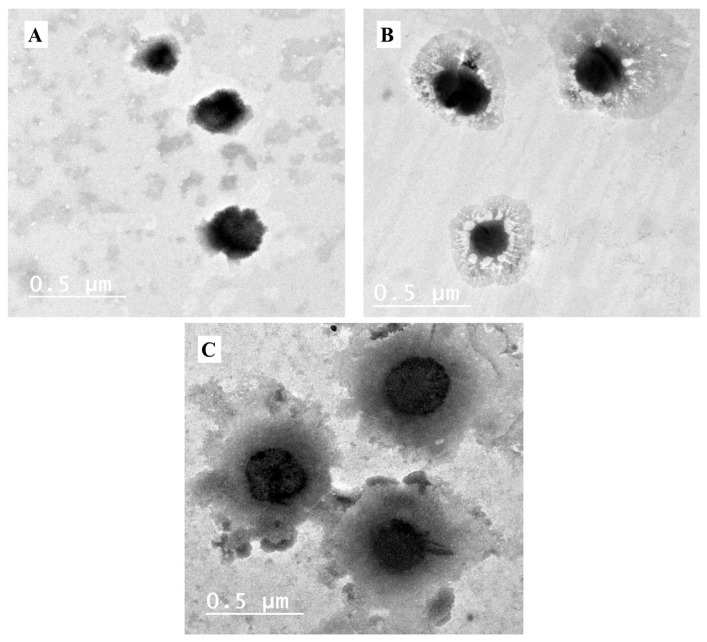
Transmission Electron Microscope micrographs of APX-NLC (**A**), APX-PEG-NLC (**B**), and APX Ch-NLC (**C**).

Representative TEM micrographs of the elaborated NLC formulations are illustrated in Figure 3. Spherical particles of a nanosized diameter can be seen in the micrographs. It is crucial to emphasize that the generated particles had a high degree of homogeneity in particle size distribution, as evidenced by the reported PDI (Table 1). Particles of APX-PEG-NLC had a more spherical form than those of APX-NLC, consistent with previously reported data [70]. Regarding chitosan-modified NLCs, a layer around the particles could be due to a drug-enriched core surrounded by chitosan surface coating [71].

#### 3.1.5. Differential Scanning Calorimetry

**Figure 4:**
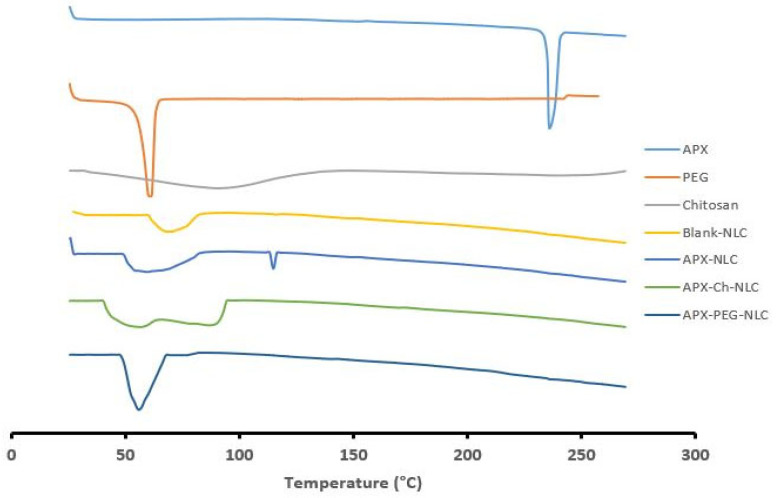
DSC thermograms of APX, PEG, Chitosan, Blank NLC, APX-NLC, APX-Ch-NLC, and APX-PEG-NLC (from up to down).

DSC was utilized to confirm the pharmaceuticals’ effective encapsulation into nanovesicles, as well as to identify interactions between medications and excipients [72]. The DSC thermograms of APX, PEG-4000, chitosan, Blank-NLCs, APX-NLC, APX-PEG-NLC, and APX-Ch-NLC are depicted in Figure 4. Pure APX showed a sharp endothermic peak at 236 °C, showing the drug’s presence in pure crystalline form. The melting endotherms of 61.5 °C and 70 °C were obtained for PEG-4000 and blank-NLCs, respectively. A broad endothermic peak between 50 °C and 130 °C was observed for chitosan, which could be attributed to the evaporation of water molecules present within the chitosan’s structure [64].

These data proved the incorporation of APX within the nanocrystalline structure of lipids, which led to increased imperfections in the lipid crystal lattice and subsequently resulted in lower melting points for the NLC formulation compared to blank-NLCs. Compared to blank-NLCs, the APX-NLC calorimetric profile showed a large endothermic peak, corresponding to the mixture of lipids, with a lower melting point than that observed for drug-free NLCs. These findings are consistent with previously reported observations for other NLC systems [73,74]. Moreover, the peak of APX at 236 °C was shifted to the lower melting point with a sharp decrease in enthalpy, indicating the conversion of the crystalline structure into a less ordered form. Regarding the PEGylated NLCs, the peaks of PEG as well as the lipid matrix were combined and shifted to a lower melting point. This melting point depression, which is often seen when a substance dissolves in another substance, suggested good miscibility of the constituents and can be ascribed to the PEG’s capacity to lower the lipids’ crystallinity [75]. The Thermogram of APX-Ch-NLC showed a bimodal endothermic peak at a lower temperature than pure chitosan and blank-NLCs. This could be due to lipid-melting and water loss from hydrophilic groups of chitosan [76]. The complete absence of an APX peak in thermograms of APX-PEG-NLC and APX-Ch-NLC indicated the dispersion of the drug in the nanoformulations as well as its physical conversion into the amorphous state, which may have enhanced the drug solubility [77].

### 3.2. Evaluation of Stability Parameters for the APX-Loaded Nanovesicles

**Figure 5:**
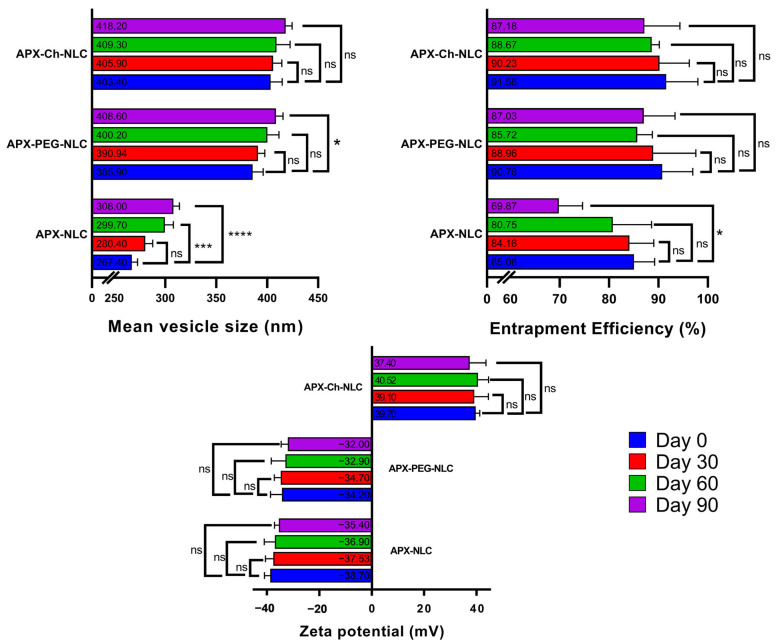
Variations in values of mean vesicle size, entrapment efficiency, and ZP over three months for APX-NLC, APX-PEG-NLC, and APX-Ch-NLC. Data have been presented as mean ± SD (n = 3). Note: *, ***, and **** denotes significant differences with *p* < 0.05, 0.001, and 0.0001, respectively, while ns denotes a nonsignificant difference between the measured parameters.

One of the essential product characteristics was its physical stability during the storage period. During 90 days of storage at 4 °C, the stability of the NLC formulations was assessed by measuring the vesicle size, ZP, and EE% (Figure 5). Studies on Day 30, Day 60, and Day 90 were conducted and compared to Day zero stability parameters results. For all nanovesicles, an increasing trend of nanocarrier size alongside the decreasing trend of both ZP and EE% was observed. The initial mean vesicle sizes of APX-NLC, APX-PEG-NLC, and APX-Ch-NLC were 267.4 ± 5.5 nm, 385.9 ± 10.2 nm, and 403.4 ± 11.5 nm, respectively. These values were changed to 308 ± 6 nm, 408.6 ± 6.9 nm, and 418.2 ± 6.4 nm, respectively, after 90 days of storage at 4 °C. After applying two-way ANOVA statistical analysis with Tukey’s multiple comparisons, our results revealed that APX-NLC had a significant increase in mean vesicle size after 60 and 90 days. The primary cause of the observed results was the coagulation of the nanocolloidal dispersion.

Regarding PEGylated NLCs, there was a nonsignificant increase in size after 60 days and a significant increase after 90 days. On the other hand, APX-Ch-NLC showed an insignificant increase in the mean vesicular diameter throughout the study period (Figure 5). The observed growth rate of the PEGylated and chitosan-coated NLCs was found to be lower than the unmodified NLCs. The presence of an additional PEG or chitosan layer provided the modified NLC formulations with additional steric stability, which reduced the coagulation of vesicles and eventually led to a reduction in growth rate [21,25]. Moreover, chitosan could increase repulsive forces among the positively charged surfaces of the NLC vesicles [78].

ZP was found to be altered from its initial values of −38.7 ± 2.2 mV, −34.2 ± 4.4 mV, and 39.7 ± 1.5 mV for APX-NLC, APX-PEG-NLCs, and APX-Ch-NLC, respectively, to −35.5 ± 1.7 mV, −32 ± 2.6 mV, and 37.4 ± 6.2 mV, respectively, after 3 months. Although the ZP may have decreased over the study time, this change was insignificant, and ZP values were found to be high enough to maintain sufficient electrostatic repulsion, which can still prevent particle aggregation.

Regarding EE%, Figure 5 shows that the entrapped drug percent decreased significantly from 85.08 ± 4.14% on Day 0 to 69.87 ± 4.76% on Day 90 for the APX-NLC formulation. The values of EE% for APX-PEG-NLC and APX-Ch-NLC during the same period changed from 90.78 ± 6.2% and 91.58 ± 6.4% to 87.03 ± 6.33 and 87.18 ± 7.17%, respectively. This nonsignificant decrease in the entrapped APX in the case of modified NLCs was due to the presence of the coating, which could have hindered the encapsulated drug’s leakage [25]. In general, the stability study outcomes for all tested NLC formulations remained within the permitted ranges, even though modified NLCs have better outcomes, indicating the crucial role of PEG and chitosan coating in enhancing the stability of formulated nanovesicles.

### 3.3. In Vivo Characterization of Developed Nanovesicles

#### 3.3.1. Pharmacokinetic Study

**Figure 6:**
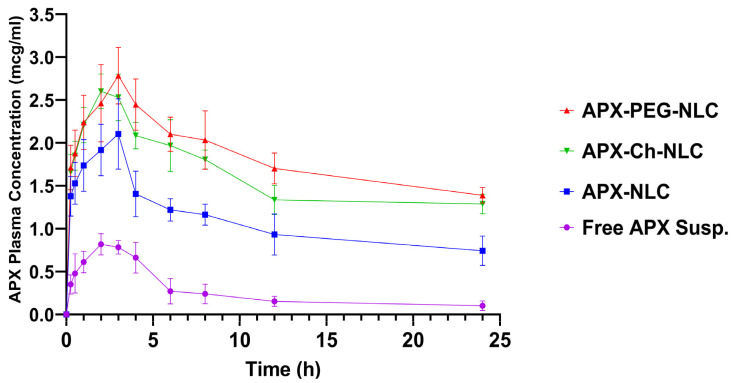
Plasma APX concentration versus time profiles following a single 60 mg/kg oral dose of APX suspension, APX-NLC, APX-PEG-NLC, and APX-Ch-NLC to male Wistar rats. Data are represented as mean ± SD (n = 3).

**Figure 7:**
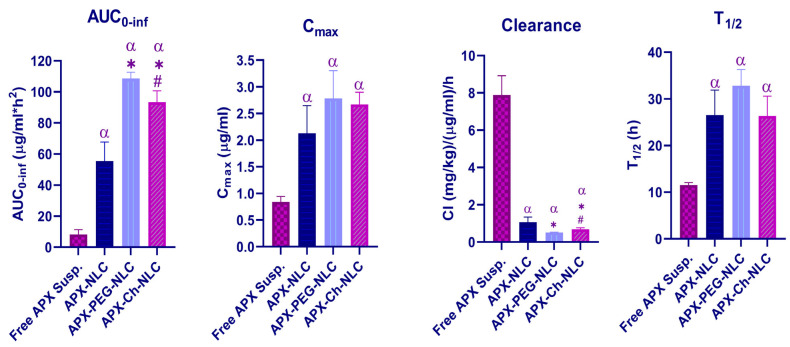
Statistical analysis of main PK parameters following oral administration of 60 mg/kg of APX suspension, APX-NLC, APX-PEG-NLC, and APX-Ch-NLC, data presented as mean ± SD (n = 3). Note: α, *, and # denote significant differences (at *p*-value < 0.05) from Free APX suspension, APX-NLC, and APX-PEG-NLC, respectively.

Following oral administration of APX-NLC, APX-PEG-NLC, APX-Ch-NLC, and free drug suspension to Wistar rats, the plasma concentration–time curve profiles were obtained and have been illustrated in Figure 6. Table 3 presents the corresponding PK parameters, and the most influencing parameters have been illustrated in Figure 7. The area under the concentration–time curve (AUC_0-inf_), following oral administration of APX-NLC, APX-PEG-NLC, and APX-Ch-NLC formulations was found to have increased by 6.7-, 13-significantly, and 11.25-folds, respectively, compared to APX-suspension. Additionally, a significant 2.5-, 3.3-, and 3.18-fold increase in C_max_ was observed for APX-NLC, APX-PEG-NLC, and APX-Ch-NLC formulations, respectively, compared with a drug suspension. These findings indicated the enhanced bioavailability of APX following oral administration of NLC formulations. This could be due to the large surface area resulting from the decreased vesicular size of NLCs. Thus, lymphatic circulation absorbs vesicles, and first-pass metabolism is avoided [20]. In addition, the presence of Tween 80 in the NLC formulations inhibits the P-glycoprotein efflux mechanism; thus, more drug is transported through the intestinal mucosa [79]. Moreover, surfactants present in the NLC formulations act as permeability enhancers and could increase membrane fluidity, leading to enhanced intestinal permeability [80]. Finally, stearic acid, a long-chain fatty acids lipid, enhances the NLC formulations’ lymphatic uptake [81]. 

Comparison between the modified and unmodified nanocarriers revealed that PEGylation and chitosanization significantly increased AUC_0-inf_ by 1.96- and 1.7-folds, respectively, relative to unmodified NLC (Table 3). The enhanced oral bioavailability of APX-NLC after PEGylation could be due to increased NLC uptake through the gastrointestinal tract and the sustained release character of PEGylated NLCs. This led to an increased in vivo retention time and, correspondingly, the time available for systemic absorption [22,82]. Additionally, a previous study showed that PEGylation enhanced cellular uptake at the site of action of hydrophobic substances more efficiently than nonPEGylated nanocarriers [70]. On the other hand, chitosan was found to enhance oral bioavailability of the APX-loaded NLCs through a further decrease in the efflux of the formulation from the GIT membrane through additional inhibition of P-glycoprotein efflux transport [83] in addition to bioadhesion and permeation potential of this cationic polymer [84]. It was observed that C_max_ was increased by 1.3- and 1.2-folds for APX-PEG-NLC and APX-Ch-NLC, respectively, compared to the unmodified NLCs. Surface modification of NLC with PEG showed superior enhancement in AUC than with chitosan. By contrast, the oral bioavailability of both surface-modified NLCs was better than APX-suspension and conventional APX-NLC.

Regarding elimination parameters, all nanovesicle formulations showed a significant increase in half-life (T_1/2_) and a significant decrease in clearance (Cl_T_) compared to APX-suspension. T_1/2_ was increased by 2.3-, 2.8-, and 2.28-folds, and Cl_T_ was decreased by 7.3-, 15.3-, and 11.43-folds for APX-NLC, APX-PEG-NLC, APX-Ch-NLC, respectively (Table 3). Prolonged half-life and a reduced total body clearance indicated the prolonged release behavior of the developed nanovesicles. This could be due to the adherence of NLC formulations to the gut wall, leading to prolonged residence time and, eventually, reducing drug clearance [85,86]. Surface modification (applied using the PEG-4000) showed a 1.2- and 1.23-folds increase in half-life, and a 2- and 1.5-folds decrease in clearance, compared to conventional NLCs and chitosan-coated NLCs, respectively. This might have been caused by the stealth effect of PEGylation, which protected particles from proteolytic enzymes, as well as from being rapidly cleared by the RES [87,88]. To conclude, NLC formulations produced an enhancement in the oral bioavailability and a reduction in systemic clearance of APX, with PEGylated NLCs having a superior effect over conventional and chitosan modified NLCs.

#### 3.3.2. Pharmacodynamic Study

**Figure 8:**
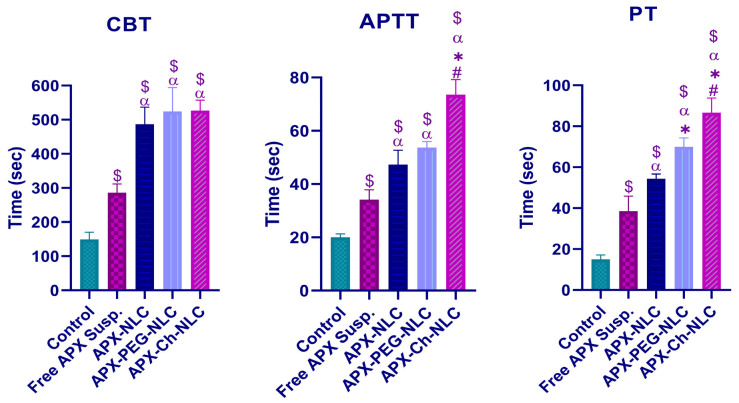
Cuticle bleeding time (CBT), activated partial thromboplastin time (APTT), and prothrombin time (PT) following administration of a single oral 60 mg/kg dose of APX suspension, APX-NLC, APX-PEG-NLC, and APX-Ch-NLC, compared to the control group. Data have been presented as mean ± SD (n = 3). Note: $, α, *, and # denote significant differences (at *p*-value < 0.05) from the control group, Free APX suspension, APX-NLC, and APX-PEG-NLC, respectively.

Bleeding and clotting time assays were performed to compare the pharmacodynamic activity of the elaborated nanoformulations to the control and free drug groups. To study hemostasis, cuticle bleeding time was used [89]. At the same time, the function of both extrinsic and intrinsic blood clotting processes was assessed by monitoring PT and APTT, respectively [90]. Figure 8 showed that APX-loaded nanoformulations significantly increased the three investigated parameters (*p* < 0.05) relative to APX suspension and control groups. Mean CBTs in the control group and the free APX suspension group were found to be 149 ± 20 and 286 ± 26 s, respectively, which increased significantly to 486 ± 50, 524 ± 70, and 526 ± 30 s for APX-NLC, APX-PEG-NLC, and APX-Ch-NLC, respectively (Figure 8). We noted that although surface-modified NLCs prolonged CBT more than conventional NLCs, this prolongation was nonsignificant.

Regarding blood clotting parameters, PT was found to be 54.33 ± 2.33, 69.9 ± 4.32, and 86.6 ± 7.2 s, with a significant increase by 1.4-, 1.8-, and 2.25-folds for APX-loaded NLC, PEGylated NLC, and APX-Ch-NLC, respectively, compared to the drug suspension. Additionally, APTT was found to be significantly prolonged by 1.38-, 1.57-, and 2.15-times for unmodified NLCs, APX-PEG-NLC, and chitosan-coated NLCs, respectively, in comparison with APX-suspension group. The influence of APX on PT was observed to be larger than that of APX on APTT, which was consistent with previously published findings [91]. The results of the PD study were in concordance with PK results, which proved the enhanced bioavailability of APX-loaded nanocarriers and, consequently, their anticoagulant efficacy. Surprisingly, the chitosan-coated NLCs produced the most significant enhancement in pharmacodynamic parameters, with prolonged PT and APTT by 1.6- and 1.55-folds, respectively, compared to unmodified NLCs, and by 1.23- and 1.37-folds, respectively, compared to PEGylated NLCs, although PEGylation was proved earlier to have the highest impact on bioavailability parameters. The reason for that could be the presence of the positively charged chitosan coating, which forms a complex with fibrinogen through hydrostatic attraction and results in conformational changes in the fibrinogen structure, thus blocking the last steps in the coagulation cascade [92].

To sum up, the different effects and mechanisms of both PEGylation and chitosanization of Apixaban-loaded NLCs are summarized in Table 4.

## 4. Conclusions

PEGylated and chitosan-coated APX-NLCs were successfully prepared and characterized in terms of vesicle size, ZP, EE%, in vitro release, TEM, and DSC, alongside a stability study, as well as further in vivo PK/PD studies. The modified nanovesicles demonstrated enhanced drug encapsulation relative to the conventional NLCs. The stability attributes of the investigated nanocarriers over a 3-month period were better retained through surface modification. The in vitro drug release pattern of APX from both PEGylated and chitosan modified NLCs was significantly sustained compared to the release profile from the nonmodified NLCs. The in vivo PK study confirmed the enhanced oral bioavailability and reduced drug clearance from both surface-modified NLCs, with PEGylation being of a superior effect than chitosan coating.

On the other hand, chitosan-coated APX-NLC had greater anticoagulant activity than APX-PEG-NLC, which could be attributed to the inactivation of fibrinogen by the chitosan, subsequently blocking the coagulation cascade. Generally, the modification of APX-NLCs surface (either with PEGylation or chitosanization) improved the physical stability, oral bioavailability, and anticoagulant activity of APX. Future work is strongly recommended to investigate the effect of the combined (mutual) PEGylation and chitosanization on the performance of APX-loaded NLCs. Proper design will be needed to determine the optimum ratios of each polymer to give the best outcome, with further research to evaluate their clinical efficacy and safety, as well as determine the optimized formulation stability.

## Figures and Tables

**Table 1:** Characterization of APX-loaded nanovesicles.

Formulations	Mean Particle Size (nm) ± SD	Polydispersity Index	ZP * (mV) ± SD	Entrapment Efficiency (%) ± SD	Cumulative Release After 24 h (%) ± SD
APX-NLC	267.4 ± 5.5	0.4	−38.7 ± 2.2	85.1 ± 4.1	82.1 ± 3.9
APX-PEG-NLC	385.9 ± 10.2	0.21	−34.2 ± 4.4	90.8 ± 6.2	72.6 ± 2.1
APX-Ch-NLC	403.4 ± 11.5	0.26	39.7 ± 1.5	91.6 ± 6.4	65.9 ± 5.7

* The difference between positive and negative ZPs was in the electrical charge of the dispersed particles of the suspension in which the ZP was measured. Thus, the positive ZP indicated that the dispersed particles in a suspension were positively charged. On the other hand, the negative ZP indicated that the dispersed particles in the suspension were negatively charged, therefore forming the difference between positive and negative ZP.

**Table 2:** Correlation coefficients (r) obtained using different kinetic release models for the APX-loaded nanovesicles.

	Zero Order	First Order	Second Order	Higuchi Diffusion Model	Hixon	Baker
APX-NLC	0.7932	−0.8336	0.8588	0.9093	0.8218	0.8386
APX-PEG-NLC	0.8388	−0.8825	0.9174	0.9387	0.8687	0.8996
APX-Ch-NLC	0.8325	−0.8659	0.8914	0.9344	0.8555	0.885

**Table 3:** Pharmacokinetic parameters of APX in male Wistar rats’ plasma following a single 60 mg/kg oral dose administration of APX suspension, APX-NLC, APX-PEG-NLC, and APX-Ch NLC. Data are expressed as mean ± SD (n = 3).

Parameter	APX Suspension		APX-NLC		APX-PEG-NLC		APX-Ch-NLC	
	Average	SD	Average	SD	Average	SD	Average	SD
K_el_ (1/h)	0.053	0.0035	0.029 ^a^	0.009	0.0217 ^a^	0.0043	0.0268 ^a^	0.0045
t_1/2_ (h)	11.56	0.493	26.55 ^a^	5.35	32.83 ^a^	3.44	26.36 ^a^	4.217
T_max_ (h)	2	NA	3	NA	3	NA	2	NA
C_max_ (μg/mL)	0.841	0.101	2.127 ^a^	0.52	2.784 ^a^	0.518	2.671 ^a^	0.225
AUC_0-t_ (μg/mL·h)	6.401	1.903	26.186 ^a^	5.053	43.99 ^a,b^	7.79	38.787 ^a,b^	2.226
AUC_0-inf_ (μg/mL·h)	8.299	3.013	55.435 ^a^	12.33	108.59 ^a,b^	4.03	93.397 ^a,b,c^	7.284
MRT_0-inf_ (h)	14.77	2.734	36.581 ^a^	12.8	47.193 ^a^	9.082	39.295 ^a^	5.512
V_d_ ((mg/kg)/(μg/mL))	145.88	50.89	40.86 ^a^	2.98	26.306 ^a,b^	6.074	25.964 ^a,b^	2.597
Cl ((mg/kg)/(μg/mL)/h)	7.89	1.03	1.069 ^a^	0.28	0.515 ^a,b^	0.016	0.6898 ^a,c^	0.085

^a^ Significantly different from values of APX suspension with *p*-value < 0.05. ^b^ Significantly different from values of APX-NLC with *p*-value < 0.05. ^c^ Significantly different from values of APX-PEG-NLC with *p*-value < 0.05. NA; not applicable.

**Table 4:** Summary of PEGylation and Chitosanization effects on APX-loaded NLCs.

	PEGylation	Chitosanization
Advantages	Significantly Enhanced Apixaban oral bioavailability over chitosanized and nonmodified NLCs through increasing AUC_0-inf_ and C_max_.	Significantly Enhanced Apixaban anticoagulant activity over PEGylated and nonmodified NLCs through increasing PT and APTT.
Mechanisms	Increased NLC uptake through the gastrointestinal tract and the sustained release character of PEGylated NLCs.	Positively charged chitosan forms a complex with fibrinogen, resulting in conformational changes of its structure and thus blocking the last steps in the coagulation cascade
Protection and stability functions	Both protected the integrity of NLC, which was confirmed by the nonsignificant decrease in the entrapment efficiency % after 90 days compared to nonmodified.	

## Data Availability

Data are available from the authors upon request.

## Supplementary Materials

The following supporting information can be downloaded at: https://www.mdpi.com/article/10.3390/pharmaceutics15061668/s1, Figure S1: Particle size distribution of APX-NLC; Figure S2. Particle size distribution of APX-PEG-NLC; Figure S3. Particle size distribution of APX-Ch-NLC; Table S1. Estimated release constants for different kinetic models of APX-loaded nanovesicles; Table S2. Korsmeyer–Peppas constants for different APX-loaded nanovesicles.
