# Supplementary material for: Influence of Surface-Modification via PEGylation or Chitosanization of Lipidic Nanocarriers on In Vivo Pharmacokinetic/Pharmacodynamic Profiles of Apixaban
Source: Pharmaceutics. 2023 Jun 7;15(6):1668. doi: 10.3390/pharmaceutics15061668 (PMC10302406; doi:10.3390/pharmaceutics15061668)
Supplement: Supplementary file 1 [file pharmaceutics-15-01668-s001.zip › pharmaceutics-2376895-supplementary.pdf]

Particle size distribution (intensity) **Hydrodynamic diameter** 267.43 nm **Polydispersity Index** 40.3%

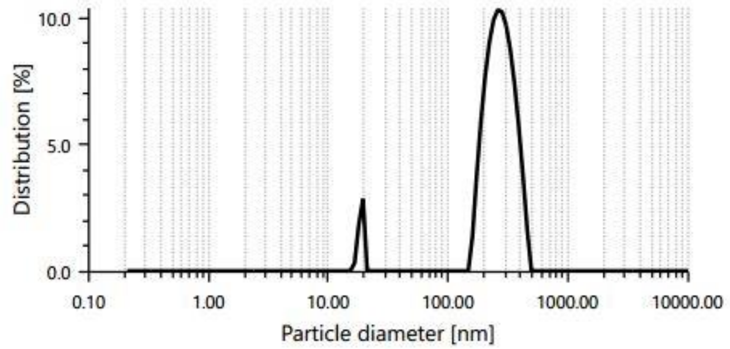

Results

**Hydrodynamic diameter**	267.4 nm	**Mean intensity**	316.3 kcounts/s
**Polydispersity index**	20.5 %	**Absolute intensity**	221445.0 kcounts/s
**Diffusion coefficient**	1.6 µm²/s	**Intercept g1²**	0.8564
**Transmittance**	52.1 %	**Baseline**	1.013

Figure S1. Particle size distribution of APX-NLC**.**

Particle size distribution (intensity) **Hydrodynamic diameter** 385.88 nm **Polydispersity Index** 20.6%

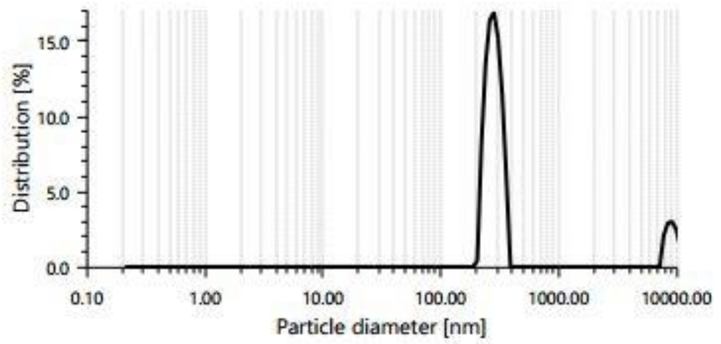

Results

**Hydrodynamic diameter**	385.9 nm	**Mean intensity**	315.0 kcounts/s
**Polydispersity index**	20.6 %	**Absolute intensity**	225612.6 kcounts/s
**Diffusion coefficient**	1.1 µm²/s	**Intercept g1²**	0.7809
**Transmittance**	52.6 %	**Baseline**	1.041

Figure S2. Particle size distribution of APX-PEG-NLC**.**

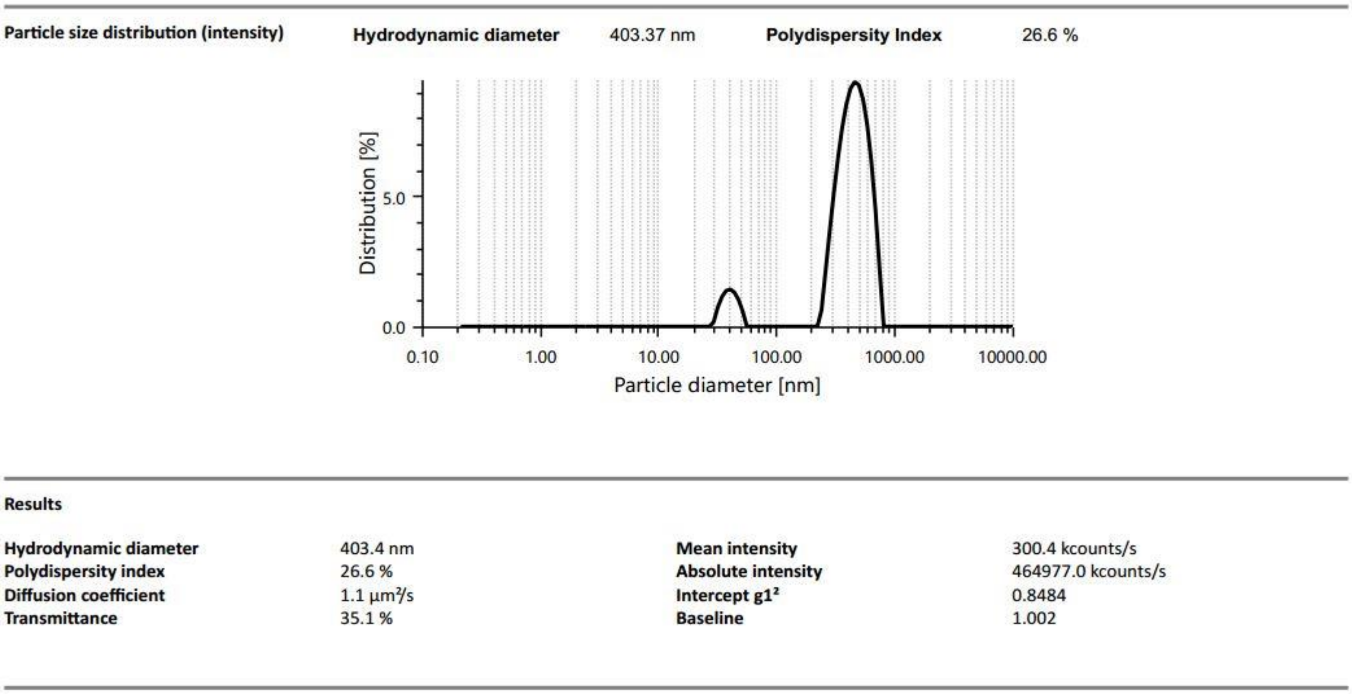

Figure S3. Particle size distribution of APX-Ch-NLC.

Table S1. Estimated release constants for different kinetic models of APX-loaded nanovesicles

Formula		Zero order	First order	Second order	Higuchi diffusion model	Hixon	Baker
**APX-NLC**	**m_0**	1.28 mg					
	**k**	2.81976	-0.0710	0.00212	18.2022	0.07923	0.00944
**APX-PEG-NLC**	**m_0**	1.36 mg					
	**k**	2.58081	-0.0522	0.0011	16.263	0.0632	0.0064
**APX-Ch-NLC**	**m_0**	1.37 mg					
	**k**	2.3612	-0.0433	0.0008	14.925	0.0543	0.005

Note: m_0 is the initial amount of drug in 3-ml sample filled in the dialysis bag (calculated according to EE% of each formulation) and k is the release rate constant for each model.

Table S2. Korsmeyer-Peppas constants for different APX-loaded nanovesicles

	APX-NLC	APX-PEG-NLC	APX-Ch-NLC
**n**	0.48955	0.51157	0.5062
**K_{kp}**	0.0312	0.0225	0.0214

Note: n is the diffusional exponent (n ≤ 0.5: Fickian diffusion, n = 0.5-1: Non-fickian release.) and K_{kp} is the release rate constant for Krosmeyer-Peppas model.
